# Integrated Pulmonary Severity Score (IPSS) for COPD: A Psycho-Respiratory Risk Index Supported by Explainable Machine Learning

**DOI:** 10.3390/diagnostics16040507

**Published:** 2026-02-07

**Authors:** Iulian-Laurențiu Buican, Alina-Catalina Buican-Chirea, Dumitru Radulescu, Ion Udristoiu, Victor Gheorman, Dragos-Mihai Cojocaru, Costin-Teodor Streba

**Affiliations:** 1 U.M.F. Doctoral School Craiova, University of Medicine and Pharmacy of Craiova, 200349 Craiova, Romania; 2 Leamna Pulmonology Hospital, 207129 Leamna, Romania; 3 Experimental Research Centre for Normal and Pathological Aging, University of Medicine and Pharmacy of Craiova, 200349 Craiova, Romania; 4 Department of Surgery, The Military Emergency Clinical Hospital ‘Dr. Stefan Odobleja’ Craiova, 200749 Craiova, Romania; 5 Department of Psychiatry, University of Medicine and Pharmacy of Craiova, 200349 Craiova, Romania; ion.udristoiu@umfcv.ro (I.U.);; 6 Sanacare AG, Schützenstrasse 1, 8401 Winterthur, Switzerland; 7 Department of Pulmonology, University of Medicine and Pharmacy of Craiova, 200349 Craiova, Romania

**Keywords:** chronic obstructive pulmonary disease (COPD), Integrated Pulmonary Severity Score (IPSS), severity assessment, anxiety, depression, explainable artificial intelligence

## Abstract

**Background/Objectives**: In chronic obstructive pulmonary disease (COPD), forced expiratory volume in one second (FEV_1_) explains only part of the variability in symptoms and prognosis, while anxiety and depression are common but rarely quantified in composite indices. We aimed to develop and internally validate an Integrated Pulmonary Severity Score (IPSS) that combines respiratory function, symptom burden and affective status. **Methods**: In a prospective observational study, 390 adults with spirometry-confirmed COPD were consecutively enrolled at two tertiary Romanian centres (October 2022–September 2024). Within 48 h of admission, patients underwent spirometry (FEV_1_% predicted), dyspnoea grading (mMRC), symptom assessment (CAT), affective evaluation (HADS-Anxiety/Depression) and cognitive screening (MoCA, MMSE). A PulmoScore was built from CAT, mMRC and ventilatory deficit (100 − FEV_1_%) and extended with a HADS-based psychiatric multiplier to obtain IPSS. Spectral clustering, logistic regression, a multilayer perceptron (MLP) and LIME were used for phenotyping and validation. **Results**: Spectral clustering identified two phenotypes—psycho-respiratory and predominantly respiratory—with acceptable separation (silhouette coefficient 0.26). The psycho-respiratory group showed lower FEV_1_%, higher CAT and mMRC scores, more severe anxiety–depression and markedly higher IPSS values. Logistic regression and the MLP achieved an accuracy of 0.89, an AUC of 0.95 and Cohen’s κ ≥ 0.75 for identifying this phenotype when using the same core clinical variables that informed phenotyping and IPSS construction. IPSS values were distributed across four strata (<30, 30–69, 70–119, ≥120 points), reflecting progressively worse respiratory and affective burden. **Conclusions**: In this cohort, IPSS captured a clinically meaningful psycho-respiratory phenotype and improved integrated severity assessment beyond spirometry alone, with potential utility for risk stratification. It can be computed from routine measures, is compatible with explainable AI workflows and warrants external, longitudinal validation before widespread implementation.

## 1. Introduction

Chronic obstructive pulmonary disease (COPD) remains among the foremost causes of global mortality and morbidity. The World Health Organization estimates that, in 2021, COPD was responsible for approximately 3.5 million deaths—around 5% of all deaths worldwide—ranking as the fourth leading cause of death [1]. Beyond its epidemiological burden, COPD is increasingly conceptualized as a biologically heterogeneous, immune-mediated inflammatory disorder in which airway damage and systemic effects are shaped by complex crosstalk between epithelial injury, innate immune activation and vascular inflammation. Recent work has highlighted cigarette-smoke–driven epithelial–immune communication through extracellular vesicles; for example, exosomal signalling has been shown to promote macrophage M1 polarization and inflammatory cell death pathways via TREM-1–related mechanisms in experimental COPD models [2]. In parallel, microRNA-regulated sirtuin axes have been implicated in cigarette smoke–induced lung injury and functional impairment (e.g., miR-132 targeting SIRT1/FoxO1) [3], while activation of SIRT6 has been reported to suppress NF-κB–driven endothelial inflammatory responses relevant to pulmonary microvascular inflammation [4]. Clinically, immune dysregulation may also translate into altered susceptibility to immune-related adverse events; notably, COPD has been associated with a higher risk of autoimmune thyroiditis during pembrolizumab therapy in non-small cell lung cancer [5]. Together, these mechanistic and clinical data support the need for integrative severity approaches that go beyond spirometry alone and reflect the multidimensional burden of COPD. Independent analyses report over 212 million people affected and more than 3 million deaths annually, elevating COPD to third place in the global mortality hierarchy [6,7]. This burden is especially acute in low- and middle-income countries, where nearly 90% of premature deaths (<70 years) occur [1].

Despite its clinical significance, commonly used composite indices—BODE, DOSE, ADO and B-AE-D—evaluate disease severity predominantly through somatic parameters, focusing on airflow limitation, exercise capacity, body mass index or exacerbation history. Yet, a substantial body of evidence demonstrates that anxiety and depressive disorders are not mere comorbidities but clinically consequential modifiers of COPD trajectories. Beyond symptom amplification, affective burden has been associated with higher healthcare utilization and poorer prognosis, including higher exacerbation risk and increased likelihood of hospital admission, and has been linked to adverse outcomes in longitudinal and registry-based studies [8,9,10,11,12,13].

From a mechanistic standpoint, anxiety intensifies the perception of dyspnoea through interoceptive hypersensitivity [14] and heightened sympathetic activation, regardless of obstruction severity [15,16,17,18]. CAT scores are significantly higher in patients with anxiety or depression, even in the absence of objective FEV_1_ deterioration [19,20,21]. Clinically, this emotional influence has been associated with a markedly increased risk of subsequent exacerbation and a self-reinforcing cycle of functional decline, disproportionate symptom burden, and escalated care needs [13,22].

In a preliminary study, our team applied k-means clustering to a comprehensive set of cognitive, affective and respiratory variables in patients with chronic respiratory disorders (asthma or COPD), identifying three distinct clusters; one of these—corresponding to a “Severe Cognitive Respiratory” profile—was dominated by marked anxiety–depressive symptoms and cognitive impairment in the context of substantial respiratory limitation, i.e., a pronounced psycho-respiratory burden [23]. These findings underscored the heterogeneity of COPD and highlighted the need for an index capable of quantifying both somatic and emotional dimensions concurrently.

On these grounds, the present study introduces the Integrated Pulmonary Severity Score (IPSS), a composite index combining airflow obstruction, symptomatic burden and anxiety–depressive load into a unified formula. Furthermore, we employ unsupervised spectral clustering—which has proven effective in delineating clinically meaningful phenotypes in asthma and sepsis [24,25]—to validate the existence of a discrete psycho-respiratory phenotype. The aim of this work is to propose and internally characterize IPSS as a pragmatic bedside tool for integrated severity assessment and risk stratification, while explicitly acknowledging the need for external, multi-ethnic and longitudinal validation before clinical implementation. Throughout the manuscript, “severity” refers to the integrated multidimensional burden quantified by the continuous IPSS, whereas “risk stratification” refers to the pragmatic assignment of patients into ordinal IPSS risk categories intended to guide clinical follow-up and management intensity.

## 2. Materials and Methods

This prospective observational study was conducted between October 2022 and September 2024 at two tertiary care centres in Romania: the Craiova Clinical Neuropsychiatry Hospital—Psychiatry Clinic (Park), which oversaw all psychiatric and cognitive evaluations, and Leamna Pulmonology Hospital, which performed respiratory function testing and symptom assessments. Adults (≥18 years) hospitalized with a spirometry-confirmed diagnosis of chronic obstructive pulmonary disease (COPD) were enrolled consecutively after providing written informed consent. Ethical approval was obtained from the Ethics Committee of the University of Medicine and Pharmacy Craiova (Protocol No. 196/17 February 2022) and from the local committees at both hospitals (Protocol No. 6190/14 October 2022), in accordance with the Declaration of Helsinki.

### 2.1. Inclusion and Exclusion Criteria

**Figure 1:**
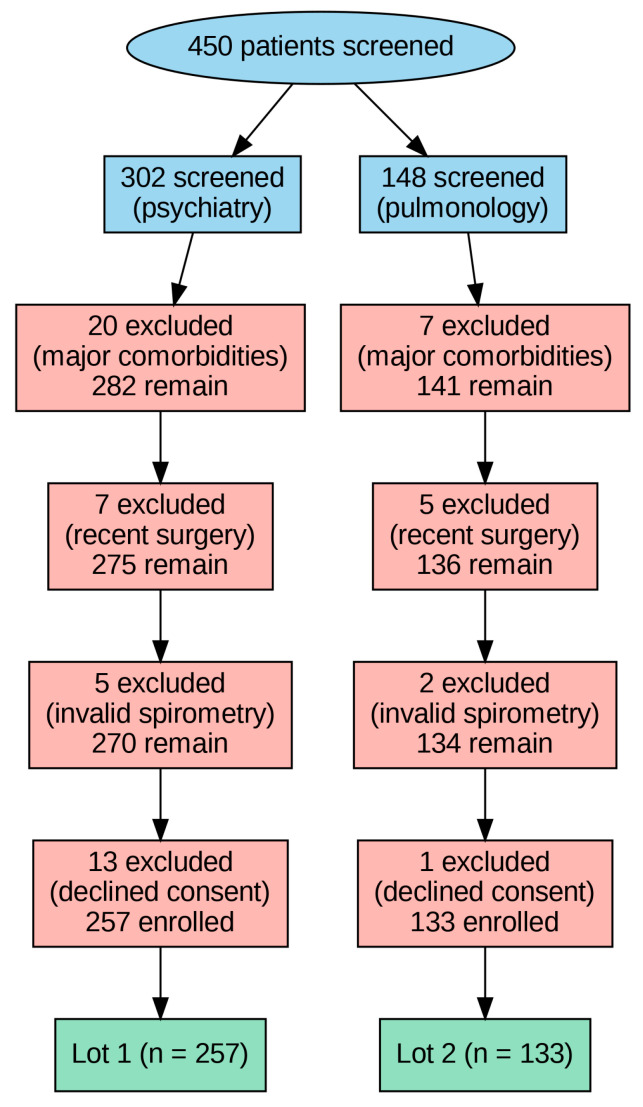
Study flowchart.

Eligible participants were adults (≥18 years) with a COPD diagnosis confirmed according to contemporary GOLD criteria who were able to provide informed consent and to complete study assessments during hospitalization. Exclusion criteria comprised major cardiometabolic comorbidities (e.g., heart failure, BMI > 35 kg/m^2^, obstructive sleep apnoea), uncontrolled psychiatric disorders beyond anxiety and depression, major surgery within the previous six months, inability to perform valid spirometry or refusal to participate. Of 450 patients screened, 60 were excluded for these reasons, yielding a final cohort of 390 patients (Figure 1).

These exclusions were applied to minimize major non-pulmonary confounding during exploratory score derivation, acknowledging that this may reduce generalizability to multimorbid real-world COPD populations.

### 2.2. Respiratory Assessment

At Leamna Pulmonology Hospital, respiratory evaluation included:

- Spirometry (Spirolab IV): FEV_1_ and FVC were measured, and predicted FEV_1_% was calculated according to reference equations; airflow limitation was subsequently graded into GOLD I–IV categories.
- mMRC Dyspnoea Scale: graded 0–4 according to dyspnoea severity during usual daily activities.
- COPD Assessment Test (CAT): eight items scored 0–5 (total 0–40) covering cough, sputum, chest tightness, activity limitation, confidence, sleep and energy. In this study, values < 10 were considered low impact, 10–20 moderate, 21–30 high and >30 very high impact.

### 2.3. Psychiatric and Cognitive Evaluation

Within 48 h at Craiova Clinical Neuropsychiatry Hospital—Psychiatry Clinic (Park), participants completed:

- Hospital Anxiety and Depression Scale (HADS): two subscales (0–21) for anxiety and depression (0–7 normal; 8–10 mild; 11–14 moderate; 15–21 severe).
- Montreal Cognitive Assessment (MoCA): 0–30 scale (26–30 normal; 18–25 mild impairment; 10–17 moderate; <10 severe).
- Mini–Mental State Examination (MMSE): 0–30 scale (25–30 normal; 21–24 mild; 10–20 moderate; <10 severe).

### 2.4. IPSS Construction and Clustering

The Integrated Pulmonary Severity Score (IPSS) was constructed in two steps. First, a pulmonary component (PulmoScore) was defined deterministically from CAT, mMRC (rescaled ×10 to harmonize with the CAT 0–40 range) and ventilatory deficit (100 − FEV_1_%), yielding an explicit weighted combination of these three variables. Second, the PulmoScore was multiplied by a psychiatric amplification factor derived from HADS-Depression and HADS-Anxiety, yielding:

IPSS = PulmoScore × [1 + (α × HADS-Depression) + (β × HADS-Anxiety)],

where α and β represent per-point fractional increases in the multiplier (cohort-derived coefficients). Coefficients and cut-offs are therefore subject to external recalibration in independent validation cohorts. Although cognitive assessments (MoCA and MMSE) were collected and analyzed to characterize phenotype burden, they were intentionally not incorporated into the final IPSS to preserve routine feasibility and portability across settings, given that cognitive testing is not universally performed at each visit and may be influenced by education, language/cultural factors and examiner variability.

To explore whether IPSS components reflected clinically meaningful patterns, we applied spectral clustering to the matrix of respiratory, affective and cognitive variables. The number of clusters (k = 2–5) was examined, and the optimal solution was chosen based on the highest average silhouette coefficient and clinical interpretability, yielding two phenotypes: a psycho-respiratory and a predominantly respiratory profile.

### 2.5. Statistical Analysis

Classical statistical analyses were performed in SPSS 26.0 (IBM Corp., Armonk, NY, USA). Continuous variables were expressed as mean ± standard deviation or median (interquartile range) and compared using independent-samples *t* tests or non-parametric equivalents, as appropriate. Categorical variables were summarized as counts and percentages and compared with χ^2^ tests. Pearson correlation coefficients were computed to assess bivariate associations between FEV_1_%, CAT, mMRC, HADS, MoCA and MMSE. A two-tailed *p* < 0.05 was considered statistically significant.

Predictive modelling was conducted in Python 3.8 (Anaconda distribution; Anaconda, Inc., Austin, TX, USA)) using scikit-learn 1.2.2 (Python Software Foundation, Wilmington, DE, USA). Data were randomly split into a training set (70%) and a test set (30%), stratified by cluster membership. The following models were fitted:

- Logistic regression: to estimate linear relationships between core predictors (respiratory, affective, cognitive variables and composite scores) and membership in the psycho-respiratory phenotype.
- Multilayer perceptron (MLPClassifier): a feed-forward neural network with two hidden layers (50 and 25 neurons), rectified linear unit (ReLU) activation and Adam optimiser, to capture potential non-linear interactions.

Model performance was evaluated using accuracy, area under the receiver operating characteristic curve (AUC) and Cohen’s kappa (κ) on the test set. Local interpretability was obtained with the LIME package, which highlighted feature contributions—particularly FEV_1_% and HADS subscales—to individual predictions. Correlation networks and graphical representations (histograms, ROC curves, κ plots) were generated with matplotlib, seaborn and networkx.

## 3. Results

### 3.1. Descriptive Analysis of the Cohort

We first examined the clinical and demographic profile of the full cohort (*n* = 390) to establish a foundation for subsequent subgroup comparisons. Both continuous and categorical variables were catalogued to capture factors potentially influencing disease course, with particular attention to differences between patients with and without significant psychiatric symptoms.

#### 3.1.1. Baseline Characteristics and Distribution of Continuous Variables

Continuous measures encompassed respiratory parameters and psychocognitive assessments. The mean age was 54.5 years (range 20–87), reflecting inclusion of both young adults and those with advanced disease. Sex distribution was balanced, and urban versus rural residence was nearly equal, ensuring broad socio-demographic representation.

Symptom severity by the COPD Assessment Test (CAT) averaged 17.3 (range 1–40), indicating the presence of both minimally and severely symptomatic subgroups. The mean Medical Research Council dyspnea grade (MRC) was 2.24 (0–4), suggesting that most patients experienced at least moderate exertional limitation. Objective obstruction, measured as percent-predicted FEV_1_, averaged 68.9% (range 28–99), with a subset demonstrating severe airflow limitation.

Psychiatric symptoms, assessed by HADS, showed mean depression and anxiety scores of 7.53 (1–20) and 8.04 (1–24), respectively. Although these averages are in the mild-to-moderate range, maximum scores indicate a subset with severe emotional disturbance. Cognitive function, measured by MoCA (mean 24.3, range 5–30) and MMSE (mean 23.8, range 6–30), was generally preserved; however, low-end scores reveal that a minority face substantial cognitive impairment. Together, these data underscore a highly heterogeneous cohort in terms of pulmonary obstruction severity, psychiatric burden and cognitive performance (Table 1).

#### 3.1.2. Subgroup Comparison: Impact of Psychiatric Symptoms

To assess how psychiatric symptoms influence respiratory impairment, patients were stratified into two groups based on HADS scores and affective history. Group 1 (n = 257) comprised individuals with combined psycho-respiratory involvement, while Group 2 (n = 133) included those with predominantly respiratory disease.

Marked differences emerged between groups. Mean percent-predicted FEV_1_ in Group 1 was approximately 60%, versus over 85% in Group 2. CAT scores averaged 22.3 in Group 1—nearly threefold higher than the 7.7 observed in Group 2—reflecting significantly heightened symptom perception among those with psychiatric comorbidity. HADS depression and anxiety scores were also significantly elevated in Group 1, and cognitive testing yielded lower MoCA and MMSE scores. A *t* test of the Integrated Pulmonary Severity Index (IPSS) produced a t-statistic of 18.33 (*p* < 0.001), confirming highly significant intergroup differences (Table 2).

#### 3.1.3. Distribution of Categorical Variables and Between-Group Comparisons

We further examined categorical variables to complete the demographic and clinical picture. In the overall cohort, 207 patients were male (53%) and 183 were female (47%); 216 lived in urban areas (55%), 174 in rural (45%); occupational status was 42% unemployed, 53% employed and 5% retired; education level spanned 54% for lower secondary, 26% for high school and 19% for higher education; 18% presented via the emergency department versus 82% in outpatient settings; smoking status was 42% never-smokers, 28% ex-smokers and 30% current smokers; alcohol use was 52% abstinent, 39% occasional and 9% chronic.

Comparisons between Group 1 and Group 2 revealed significant differences in several categories (χ^2^ test): sex distribution (*p* = 0.050), urban versus rural residence (*p* = 0.020), unemployment rate (*p* = 0.010), education level (*p* = 0.005), mode of presentation (*p* = 0.030), smoking status (*p* = 0.010) and alcohol consumption (*p* = 0.020) (Table 3).

These findings suggest that socio-demographic factors and health behaviours may modulate the interplay between psychiatric comorbidity and respiratory severity.

### 3.2. Analysis of Relationships Between Variables: Correlations and the Network of Interdependencies

**Figure 2:**
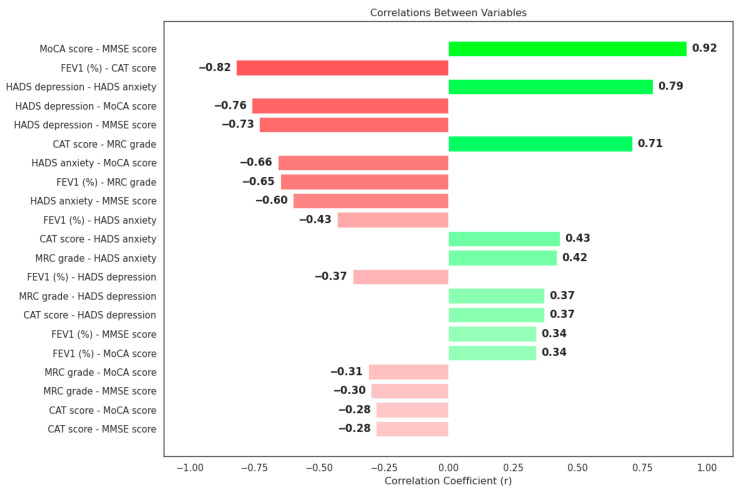
Correlations between clinical and psychological variables (r coefficient), (Green bars indicate positive correlations, while red bars indicate negative correlations).

We quantified pairwise associations among respiratory measures (percent-predicted FEV_1_, CAT and mMRC scores), psychiatric indices (HADS-Depression and HADS-Anxiety) and cognitive assessments (MoCA and MMSE) using Pearson’s correlation coefficient. A very strong inverse correlation emerged between percent-predicted FEV_1_ and CAT score (r = −0.82, *p* < 0.001) and between FEV_1_ and mMRC grade (r = −0.65, *p* < 0.001), while CAT and mMRC were strongly interrelated (r = 0.71, *p* < 0.001), confirming that declining lung function is tightly linked to worsening symptom burden and dyspnoea (Figure 2).

MoCA and MMSE also showed an almost perfect positive correlation (r = 0.92, *p* < 0.001), supporting the consistency of the cognitive assessments.

HADS-Depression and HADS-Anxiety scores correlated moderately and positively with both CAT and mMRC (r ≈ 0.37–0.43, all *p* < 0.001), indicating that affective burden contributes to, but does not fully explain, perceived respiratory symptoms. By contrast, HADS scores showed strong negative correlations with cognitive performance—for example, r = −0.76 between HADS-Depression and MoCA and r = −0.73 with MMSE—together with a strong positive correlation between HADS-Depression and HADS-Anxiety themselves (r = 0.79, *p* < 0.001). These patterns suggest that elevated affective symptoms are accompanied by measurable cognitive decline, which may impede treatment self-management (Table 3).

To illustrate these interdependencies, we constructed a correlation network in which each node represents one of the seven key variables and edges denote significant correlations (|r| > 0.30, *p* < 0.05). This network revealed a central hub linking FEV_1_%, CAT and mMRC directly to HADS-Depression and HADS-Anxiety, which in turn showed inverse ties to MoCA and MMSE. Clinically, this topology suggests that losses in FEV_1_ may be perceived as disproportionately severe in patients with concurrent anxiety or depression—driving up CAT and mMRC scores—while those psychiatric symptoms are themselves associated with reduced cognitive capacity, potentially complicating disease management.

Taken together, these findings substantiate a shared core of physiological and psychological factors—where respiratory impairment, affective distress and cognitive decline are tightly interwoven—providing a robust multidimensional rationale for an integrated severity index such as IPSS.

### 3.3. Unsupervised Clustering and Natural Subgroup Separation

To explore whether the data naturally partition into two discrete subgroups—independently of the HADS-based classification—we applied spectral clustering to a set of standardized variables encompassing FEV_1_% predicted, CAT score, MRC dyspnoea grade, HADS depression and anxiety scores, and cognitive performance as assessed by MoCA and MMSE. Standardization ensured that each metric contributed equally to the multidimensional analysis, eliminating scale-related biases.

We assessed cluster validity using the Silhouette score, which identified an optimal solution at k = 2. In other words, the high-dimensional data define two well-separated regions, each corresponding to a cohort of patients with similar clinical profiles. Notably, the resulting clusters showed marked concordance with our pre-defined HADS groups: “Cluster 1” comprised predominantly patients with combined psychiatric and respiratory impairment, whereas “Cluster 2” aligned largely with those exhibiting primarily respiratory dysfunction.

This convergence between an unsupervised clustering approach and the HADS-driven classification further substantiates the hypothesis that psychiatric factors—particularly anxiety and depression—together with respiratory metrics (CAT, MRC, FEV_1_) constitute a core determinant of natural subgroup separation. Thus, beyond confirming the existence of two distinct phenotypic clusters, our analysis underscores the importance of integrating psychiatric assessment into the evaluation of pulmonary disease severity.

In summary, the intrinsic bifurcation of patient subgroups revealed by spectral clustering—and validated by the Silhouette Index—reinforces the notion that emotional symptoms can amplify both the perception and impact of respiratory impairment, providing a solid foundation for the future development of an integrated severity index.

### 3.4. Classification Models and Clinical Validation

To determine how well our predictors identify patients with combined psychiatric–respiratory impairment (Cohort 1), we compared two approaches: a logistic regression and a multi-layer perceptron (MLP) neural network. Both models were trained on the same feature set—HADS depression and anxiety scores, respiratory measures (CAT, FEV_1_%, MRC dyspnoea grade) and global cognition (MoCA, MMSE).

**Figure 3:**
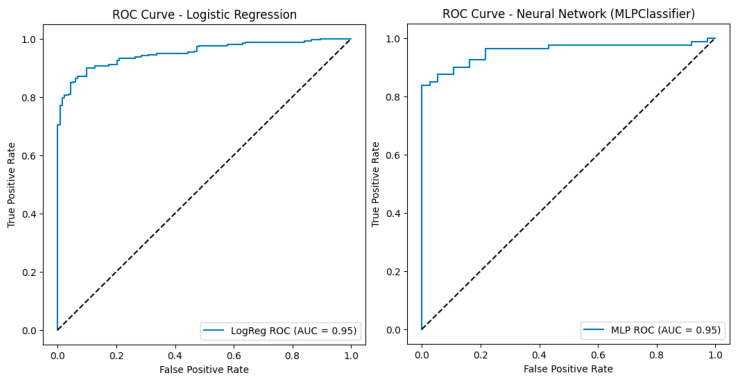
ROC Curves for logistic regression and MLPClassifier.

**Figure 4:**
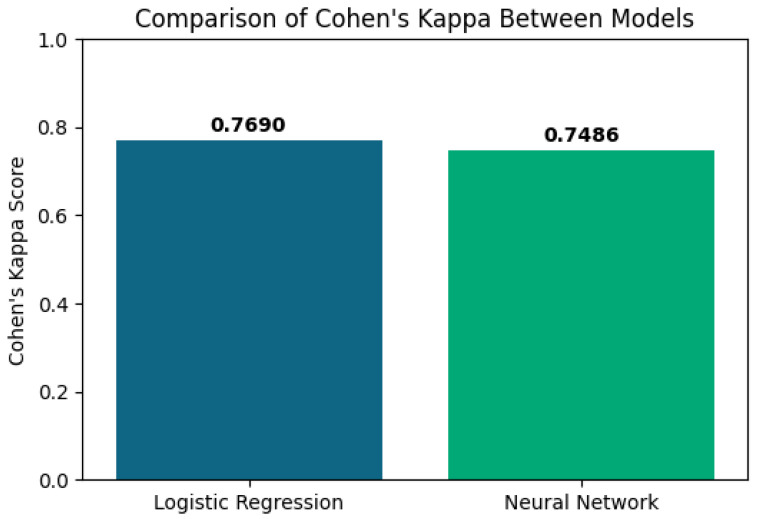
Comparison of Cohen’s kappa scores between logistic regression and the neural network.

The logistic regression achieved 89% accuracy, an area under the ROC curve (AUC) of 0.95 (Figure 3) and Cohen’s κ of 0.77 (Figure 4).

Regression coefficients highlighted CAT score—and above all HADS-Anxiety—as the strongest positive predictors of Cohort 1 membership, indicating that rising anxiety and symptom burden dramatically increase the odds of dual impairment. By contrast, FEV_1_% exerted a protective effect (negative coefficient), implying that higher lung function attenuates the risk of psychiatric–respiratory comorbidity. Together, these coefficients frame a clear narrative: as psychiatric symptoms intensify and pulmonary function declines, clinical complexity escalates.

In parallel, an MLPClassifier with two hidden layers (50 and 25 neurons) yielded virtually identical performance—89% accuracy, AUC 0.95 and Cohen’s κ of 0.75 (Figure 4).

**Figure 5:**
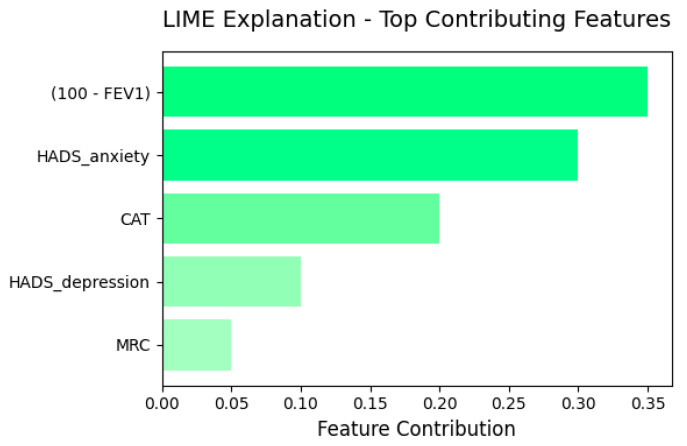
Feature contribution to the model decision according to LIME.

To probe local decision drivers, we applied LIME, which consistently flagged low FEV_1_% and elevated HADS-Anxiety as the dominant features (Figure 5).

These findings reinforce the notion that, beyond isolated measurements, the interplay between psychiatric symptomatology and airflow obstruction constitutes a powerful predictor of clinical severity. A unified insight from both classification frameworks is that psychiatric manifestations—most notably anxiety—and declining pulmonary function (as reflected by a low FEV_1_%) operate in concert to define disease burden. As anxiety and depression worsen and lung function deteriorates, the risk of adverse outcomes rises, underscoring the imperative for a multidisciplinary approach in patients exhibiting dual impairment.

**Figure 6:**
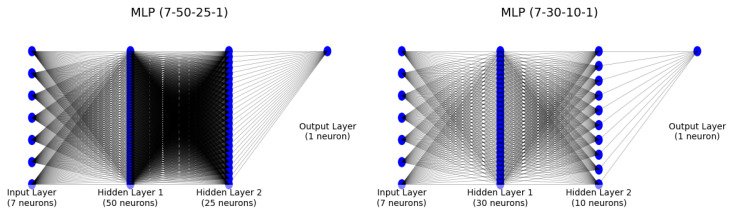
Graphical representation of two MLP architectures: (7-50-25-1) vs. (7-30-10-1).

To offer further visual contrast between modelling strategies, Figure 6 depicts the architecture of the MLPClassifier—highlighting its two hidden layers (50 and 25 neurons)—against the single-layer, unidirectional structure of logistic regression.

This schematic illustrates the added complexity of the neural network and its capacity to capture non-linear data relationships, which underpins its performance parity with the more interpretable regression model.

In sum, both logistic regression and neural-network approaches confirm that psychiatric distress and airflow limitation, particularly low FEV_1_%, are decisive in characterizing integrated disease severity. This underscores the value of integrating psychiatric evaluation into routine clinical assessment to support prognostic risk stratification and guide therapeutic decision-making.

### 3.5. Development and Implementation of the Integrated Pulmonary Severity Score (IPSS)

Given the strong correlations between FEV_1_%, symptom burden, dyspnoea, affective scores and cognition (e.g., r = −0.82 for FEV_1_% vs. CAT, r = −0.65 for FEV_1_% vs. mMRC, r = −0.43 and −0.37 for FEV_1_% vs. HADS-Anxiety and HADS-Depression, respectively, all *p* < 0.001), we sought to condense this multidimensional information into a single, clinically usable index. To this end, we constructed the Integrated Pulmonary Severity Score (IPSS) in two steps: first, by defining a pulmonary component that integrates obstruction and respiratory symptoms, and second, by applying a psychiatric multiplier reflecting the additional burden of anxiety and depression.

#### 3.5.1. Construction of the Pulmonary Component (PulmoScore)

The pulmonary component, termed the PulmoScore, was defined a priori from three clinically interpretable variables: CAT, mMRC and ventilatory deficit expressed as (100 − FEV_1_%). To harmonize scales, mMRC (0–4) was rescaled by multiplying by 10 to align with the CAT 0–40 range. The PulmoScore was then computed deterministically as:

PulmoScore = 0.30 × CAT + 0.30 × (mMRC × 10) + 0.40 × (100 − FEV_1_%).

A slightly higher weight was assigned to ventilatory deficit to anchor the score to objective obstruction, while CAT and rescaled mMRC contribute equally to capture patient-reported symptom burden and dyspnoea-related functional limitation. Under this formulation, a 10-point decrease in FEV_1_% increases the PulmoScore by 4 points, whereas a 10-point increase in CAT or a 1-grade increase in mMRC (i.e., +10 after rescaling) increases the PulmoScore by 3 points.

#### 3.5.2. Addition of the Psychiatric Multiplier and Definition of IPSS

To capture the contribution of affective symptoms to overall disease severity, the PulmoScore was extended with a psychiatric multiplier based on HADS-Depression and HADS-Anxiety. The final index was defined as:

IPSS = PulmoScore × [1 + (α × HADS-depression) + (β × HADS-anxiety)]

**Figure 7:**
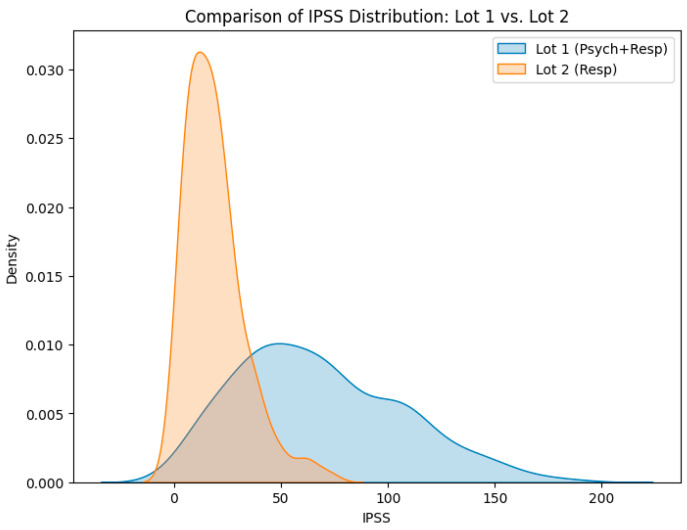
Comparative distribution of IPSS between Group 1 (blue) and Group 2 (orange).

Using ordinary least squares regression with the global psycho-respiratory index (SGPI) as the dependent variable, the coefficients were estimated as α = 0.0231 and β = 0.0423 (per-point fractional increases in the multiplier), while the intercept was small and not retained for bedside computation. Visual inspection of the final IPSS distribution (Figure 7) did not suggest marked score inflation or extreme outliers in this cohort. Thus, each additional HADS-Depression point increases the multiplier by 2.31%, and each additional HADS-Anxiety point increases the multiplier by 4.23%, holding other components constant. The larger coefficient for HADS-Anxiety should be interpreted cautiously: it is cohort-derived and may reflect stronger coupling between anxiety, dyspnoea perception and patient-reported symptom burden in this sample rather than a universal hierarchy of affective domains. Accordingly, these coefficients require external validation and may need recalibration across populations and care settings. In the cohort, this formulation produced IPSS values ranging from very low scores in patients with preserved lung function, minimal symptoms and normal HADS values to triple-digit scores in individuals with combined severe obstruction, high CAT and mMRC grades and marked anxiety–depression.

#### 3.5.3. Interpretation of the IPSS Formula

The IPSS is obtained in two conceptually simple steps. First, the PulmoScore aggregates objective and subjective respiratory information into a single pulmonary severity index. Second, this PulmoScore is multiplied by a factor that increases with HADS-Depression and HADS-Anxiety scores, thereby “amplifying” the respiratory burden in the presence of affective comorbidity.

Clinically, this behaviour is intuitive:

- Patients with relatively preserved FEV_1_%, low CAT and mMRC scores and normal HADS values tend to have low PulmoScores and remain in the lower IPSS range, corresponding to a predominantly somatic but mild overall burden.
- Patients with comparable spirometric impairment but elevated HADS-Anxiety and/or HADS-Depression exhibit substantially higher IPSS values, shifting them into higher risk strata despite similar obstruction.

This mechanism formally explains the frequent discrepancy between spirometry and perceived health status in COPD: the psychiatric multiplier captures the additional psycho-emotional load that is invisible to FEV_1_% alone. In line with this, the psycho-respiratory cluster (Group 1) was characterized by lower FEV_1_%, higher CAT and mMRC scores and markedly higher HADS values, while the predominantly respiratory cluster (Group 2) had better lung function, lower symptom burden and near-normal affective scores.

#### 3.5.4. Distribution of IPSS Values and Risk Categories

To render the IPSS interpretable at the bedside, we grouped the continuous score into four ordinal risk categories that balance the empirical score distribution with clinically intuitive cut-points:

- Low risk: IPSS < 30 points
- Moderate risk: IPSS 30–69 points
- High risk: IPSS 70–119 points
- Very high risk: IPSS ≥ 120 points.

To provide an explicit empirical reference for stratification, we also report the distribution-based cut-points of IPSS in this cohort. The IPSS quartiles were Q1 = 17.79, Q2 (median) = 35.91 and Q3 = 54.77. Accordingly, a quartile-based stratification would define four groups as IPSS ≤ 17.79, 17.79 < IPSS ≤ 35.91, 35.91 < IPSS ≤ 54.77, and IPSS > 54.77. We emphasize that both the pragmatic thresholds and the quantile-based cut-points are cohort-dependent and should be recalibrated and/or outcome-optimized in future external validation studies.

These bands were not optimized against hard endpoints (e.g., exacerbations, hospitalisations, mortality) in this cross-sectional derivation cohort. Instead, they were selected as pragmatic, distribution-informed thresholds approximating the sample quartiles and then rounded to improve bedside usability. Accordingly, these cut-offs should be recalibrated and/or outcome-optimized in external longitudinal validation cohorts before being used for prognostic decision-making. Patients were represented in all four strata, with a progressive gradient in respiratory impairment, symptom burden and affective distress from the low to the very-high IPSS category (Table 4).

Descriptively, individuals with preserved lung function, low CAT and mMRC scores and normal or near-normal HADS values were concentrated in the low and moderate IPSS categories. By contrast, those combining severe obstruction, marked dyspnoea, high CAT scores and significant anxiety–depression predominantly occupied the high and very-high bands. This gradient closely mirrored the distribution of the two spectral-clustering phenotypes: the psycho-respiratory Group 1 showed a marked right-shift in the IPSS distribution, with a higher proportion of high and very-high scores compared with Group 2, whereas Group 2 was concentrated almost entirely in the low and moderate IPSS range (Figure 7).

From a practical standpoint, these categories offer a simple decision-making framework. Low-IPSS patients are typically candidates for routine follow-up and standard pharmacological management. Moderate IPSS values signal the need to optimize inhaled therapy and initiate structured psychological support. High and very-high IPSS identify a subset requiring intensified and often multidisciplinary care, potentially including psychiatric consultation, pulmonary rehabilitation and closer monitoring.

#### 3.5.5. Relationship Between IPSS-Based Profiles and Predictive Models

The clinical relevance of this integrated construct was further supported by the performance of supervised models trained to distinguish the two spectral-clustering phenotypes. Because the clustering procedure and IPSS construction relied on overlapping core variables, these supervised results should be interpreted as convergent internal validity (consistency with the latent structure captured by clustering) rather than as an independent external validation. A logistic regression model using the core IPSS components (FEV_1_%, CAT, mMRC, HADS-Depression, HADS-Anxiety, MoCA and MMSE) as predictors correctly classified 89% of patients (accuracy 0.89), with an area under the ROC curve (AUC) of 0.95 and a Cohen’s κ of 0.77, indicating almost perfect agreement beyond chance. A multilayer perceptron (MLP) neural network trained on the same variables and evaluated on an independent test set yielded a similar accuracy of 0.89, an AUC of 0.95 and a Cohen’s κ of 0.75.

LIME explanations for the MLP underscored that low FEV_1_% and elevated HADS-Anxiety scores were the most influential features for predicting assignment to the psycho-respiratory group, followed by intermediate levels of HADS-Depression, mMRC and CAT. These findings are consistent with the design of IPSS and reinforce the notion that integrating pulmonary function, symptom burden and affective status captures a clinically meaningful psycho-respiratory phenotype rather than a purely statistical construct.

## 4. Discussion

In contemporary pulmonology practice, there is broad consensus that disease severity assessment cannot be confined to the predicted percentage of FEV_1_, although this remains an essential physiological pillar. Established instruments—BODE, DOSE, ADO and B-AE-D—have successfully correlated spirometric values with mortality, exacerbation frequency or healthcare utilization; nevertheless, they almost exclusively describe the somatic dimension and systematically omit psycho-emotional factors. The Integrated Pulmonary Severity Score (IPSS) provides a coherent conceptual extension by explicitly incorporating anxiety–depressive symptomatology as a variable with its own numerical weight, thus allowing it to enter severity models on an equal footing with spirometry and dyspnoea.

The premise for this integration arises from a robust convergence of evidence: anxiety amplifies the perception of breathlessness and dyspnoea-related fear in COPD [26,27], elevates CAT scores irrespective of the obstruction degree [19,20] and—when co-existing with depression—more than doubles the risk of future exacerbations [13]. Depression, in turn, overlaps with those neurocognitive and functional domains that drive prognosis: COPD-related cognitive impairment, frequently accompanied by depressive symptoms, has been linked to frontal lobe hypoperfusion on SPECT imaging [28] and to poorer performance on both MoCA and MMSE [29,30]. At the same time, dyspnoea severity itself—strongly intertwined with affective burden—independently predicts in-hospital mortality during acute exacerbations [31]. In light of these observations, the direct inclusion of HADS scores in a composite severity index is not merely optional but appears methodologically necessary to reflect the true impact of the disease on the patient.

### 4.1. Rationale for the IPSS Structure

The PulmoScore—the core respiratory component—aggregates the CAT, mMRC and ventilatory deficit, with a weight of 0.40 assigned to (100 − FEV_1_%). This higher weighting for obstruction derives from the observation that, in our cohort, the FEV_1_–symptom relationship (r = −0.82 for FEV_1_% vs. CAT) was considerably tighter than the modest correlations reported in multicentre CAT validation studies [32,33]. Similar observations have emerged from the COSYCONET registry, where longitudinal declines in FEV_1_ tracked worsening SGRQ scores over time [34].

A relatively greater weight for FEV_1_ is also justified by data on early-onset COPD: younger adults can exhibit marked airflow limitation and unfavourable trajectories despite relatively preserved multidimensional scores, challenging the traditional Fletcher–Peto paradigm [35,36]. Large cohort analyses show that “young COPD” patients carry a substantial burden of comorbidities and an excess mortality risk compared with age-matched ever-smoker controls [37]. In such individuals, indices that strongly weight chronological age—such as ADO—may yield deceptively low risk estimates despite clinically significant impairment. By contrast, including both FEV_1_ and patient-reported measures (CAT and mMRC) within the PulmoScore preserves the subjective dimension of symptom burden while anchoring it to the objective severity of obstruction, thus offering an immediate and clinically interpretable snapshot without requiring elaborate exercise testing.

### 4.2. Detailed Comparison with Established Indices

BODE remains a robust predictor of mortality in COPD, but it relies on the six-minute walk test (6MWT), which is difficult to standardize and is not routinely available in many outpatient clinics, limiting its practicality in everyday practice [38,39]. DOSE prioritizes exacerbation history and current smoking status, variables that can be collected rapidly in primary care and that have demonstrated prognostic value for mortality and healthcare utilization [39]. However, active smokers often underestimate or normalize respiratory symptoms and therefore underreport them, which may attenuate the sensitivity of DOSE to detect clinically relevant risk [40,41].

ADO introduces chronological age as an essential variable alongside dyspnoea and FEV_1_, yet early-onset and rapidly progressive COPD have been described in younger adults, indicating that a strong age component can still underestimate risk in susceptible individuals [35,36,37]. B-AE-D incorporates body mass index, prior severe exacerbations and mMRC dyspnoea grade, and its extended version (B-AE-D-C) adds the biomarker copeptin, whose measurement requires laboratory infrastructure that is not universally accessible [42]. By contrast, IPSS relies exclusively on spirometry and brief questionnaires, allowing calculation in 3–5 min and facilitating inter-centre comparability without additional investment. Importantly, IPSS is not proposed as a replacement for these established indices but as a complementary tool that explicitly integrates psycho-emotional burden into severity assessment. From the standpoint of everyday implementation, these instruments differ markedly in the time, infrastructure and data completeness they require. BODE offers strong prognostic performance, but its dependence on a standardized 6MWT introduces logistical barriers (space, staffing, protocol consistency) and limits rapid use in busy outpatient clinics and in telemedicine workflows. DOSE is well suited to primary care because its components can be collected quickly, yet it depends on accurate exacerbation history and current smoking status; both can be incompletely documented or variably reported, which may reduce sensitivity for clinically meaningful risk in routine practice. ADO improves prognostic discrimination by incorporating age, but a strong age component can yield deceptively low risk estimates in younger patients with marked airflow limitation or rapidly progressive disease. In contrast, IPSS was designed for maximal feasibility: it uses spirometry plus short questionnaires (CAT, mMRC, HADS) that are routinely obtainable in most settings, enabling computation within minutes and supporting digital capture in registries, spirometry software, and remote monitoring platforms. Practically, IPSS therefore complements established indices by offering a rapid, reproducible multidimensional snapshot that makes psycho-emotional burden quantitatively visible alongside obstruction and symptom severity.

### 4.3. Convergence with Unsupervised Phenotyping and Network Analysis

Applying spectral clustering to the clinical variable matrix yielded two clinically coherent clusters that showed substantial overlap with groups defined by HADS scores, suggesting that anxiety, depression, CAT, mMRC and FEV_1_ constitute a major axis of variability in this dataset [24,25]. Network-based analyses placed affective variables among high-centrality nodes [43,44], indicating not merely passive association but a potential generative role in clinical distress and functional limitation. This unsupervised convergence supports the notion that the IPSS formula approximates the latent structure of the data rather than merely modelling relationships imposed a priori. Nevertheless, because the same feature set underlies both clustering and score construction, this convergence should be viewed as supportive internal evidence, and independent validation against external cohorts and longitudinal outcomes remains required.

### 4.4. Clinical Implementation and Pragmatic Benefits

IPSS uses five routinely available variables (FEV_1_%, CAT, mMRC, HADS-Anxiety and HADS-Depression) and can be embedded into spirometry software, outpatient workflows, and telemonitoring platforms. In practice, IPSS can function as a triage and personalization aid: (i) it can flag patients who may benefit from earlier multidisciplinary review (pulmonology + psychiatry/psychology), (ii) it can serve as a structured trigger for psychological referral (including telepsychiatry in low-access regions), and (iii) it can support prioritization for pulmonary rehabilitation and structured behavioural interventions (e.g., CBT-informed programmes) aimed at reducing affective distress and improving function [45,46,47,48]. Importantly, while higher IPSS plausibly indicates increased vulnerability to exacerbation and healthcare utilization, prospective longitudinal validation against hard endpoints is required before IPSS is used as an outcome-prediction tool in routine care. Moreover, the structure of IPSS is amenable to explainable-AI pipelines: in our cohort, LIME highlighted HADS-Anxiety and FEV_1_ as leading contributors to the model output, consistent with comparative evaluations showing the usefulness of local interpretability techniques such as LIME and SHAP for distinguishing clinically meaningful classes [49,50]. These characteristics suggest that IPSS can act as a bridge between traditional bedside assessment and algorithm-assisted decision support systems.

### 4.5. Relevance to Public Health Policy

Our analysis revealed significantly higher IPSSs among patients with lower educational attainment, precarious occupational status and rural residence—findings that mirror research on the social determinants of health and on COPD-related disparities in access and outcomes [51,52]. Integrating IPSS into a national electronic registry could enable continuous monitoring of such inequalities and support dynamic resource reallocation, in line with Frohlich and Potvin’s ecosocial perspective on the “determinants of determinants” [53] and with evidence of rural disadvantage in quality of life and service availability for patients with COPD [45,54].

In summary, IPSS addresses important gaps left by traditional composite indices by offering a patient-centred tool that simultaneously quantifies ventilatory impairment and emotional burden. Its internal validation through unsupervised clustering and supervised models, its feasibility in everyday clinical workflows and its compatibility with digital infrastructure recommend IPSS as a promising candidate for individualized clinical decision-making and for health policies adapted to the current socio-epidemiological context. At the same time, external validation in independent cohorts and prospective evaluation against hard clinical outcomes (exacerbations, hospitalizations, mortality) remain essential before IPSS can be adopted as a definitive standard in COPD severity assessment.

## 5. Limitations of the Study

This study has several limitations. First, its bi-institutional design (psychiatric vs. pulmonology recruitment) may introduce selection/spectrum bias due to differences in referral patterns and the routine use of psychocognitive assessment. Second, major cardiometabolic comorbidities (e.g., heart failure, sleep apnoea, severe obesity) were excluded to reduce confounding during score derivation; however, this limits generalizability to real-world multimorbid COPD, and future studies should validate and, if needed, recalibrate IPSS in broader cohorts, ideally including sensitivity analyses with relaxed exclusions and/or comorbidity-stratified models. Third, IPSS coefficients and cut-offs were derived and internally evaluated in the same dataset; thus, discrimination of cluster membership should be interpreted as internal separability rather than independent validation, and external validation in independent, multi-ethnic cohorts is required. Fourth, the cross-sectional design precludes causal inference and does not allow assessment of longitudinal predictive accuracy against hard endpoints (exacerbations, hospitalisations, mortality or healthcare costs). Fifth, CAT, mMRC and HADS are self-reported measures and are susceptible to measurement error and interviewer/context effects, including cultural and educational influences. Finally, the absence of continuous telemonitoring data (nocturnal SpO_2_, physical activity) and biological markers limits comparisons with biomarker-enriched indices and the ability to capture rapid clinical changes; additionally, the multiplicative formulation may be sensitive to distributional shifts and should be re-assessed for skewness/outliers during external validation, while the exclusion of cognitive scales from IPSS reflects a feasibility–comprehensiveness trade-off that should be revisited in settings where standardized cognitive testing is systematically available.

## 6. Conclusions

This study demonstrates that incorporating anxiety and depression into COPD assessment adds clinically relevant information beyond spirometry and dyspnoea scales alone. By combining FEV_1_%, mMRC, CAT and HADS-based affective scores, the Integrated Pulmonary Severity Score (IPSS) captures a psycho-respiratory phenotype characterized by more severe airflow limitation, higher symptom perception and poorer cognitive performance than that seen in predominantly respiratory profiles.

In our cohort, IPSS showed good internal validity and clearly separated patients into four severity strata (<30, 30–69, 70–119, ≥120 points), which may support graded clinical decisions ranging from routine follow-up to intensified multidisciplinary management. Both logistic regression and a multilayer perceptron achieved high discrimination (AUC 0.95, Cohen’s κ ≥ 0.75) when using the same core variables that informed the clustering and IPSS construction to classify membership in the psycho-respiratory phenotype; therefore, this agreement supports internal (convergent) validity rather than independent validation, while explainable-AI analyses (LIME) consistently highlighted HADS-Anxiety and FEV_1_% as dominant contributors. These findings suggest that IPSS can complement existing indices by making the emotional burden of COPD quantitatively visible in everyday practice.

At the same time, the score was derived and tested in two tertiary centres, excluded major cardiometabolic comorbidities and lacked longitudinal endpoints such as exacerbations, hospitalisations and mortality. Before routine clinical adoption, IPSS therefore requires external validation in independent populations, potential recalibration of cut-off values and prospective evaluation of its impact on outcomes and resource allocation. If these steps confirm its performance, IPSS could help bridge the gap between physiological impairment and emotional distress in COPD and serve as a template for future multidimensional, explainable prediction models and patient-centred, interdisciplinary care strategies.

## Figures and Tables

**Table 1:** Distribution of continuous variables of the entire cohort (N = 390).

Variable	Mean ± SD	Range
Age (years)	54.5 ± 14.2	20–87
CAT Score	17.3 ± 11.0	1–40
MRC Grade	2.24 ± 1.12	0–4
FEV_1_ (%)	68.9 ± 18.5	28–99
HADS—Depression	7.53 ± 4.30	1–20
HADS—Anxiety	8.04 ± 5.35	1–24
MoCA	24.3 ± 5.02	5–30
MMSE	23.8 ± 4.72	6–30

**Table 2:** Comparison of continuous variables between Group 1 and Group 2.

Variable	Group 1 (n = 257)	Group 2 (n = 133)	*p*-Value
Age (years)	56.60 ± 12.60	50.42 ± 16.04	0.003
MoCA Score	23.02 ± 5.50	26.68 ± 2.62	<0.001
MMSE Score	22.69 ± 5.17	25.86 ± 2.64	<0.001
HADS—Depression	8.48 ± 4.53	5.69 ± 3.07	<0.001
HADS—Anxiety	9.54 ± 5.77	5.15 ± 2.63	<0.001
CAT Score	22.25 ± 9.67	7.74 ± 5.91	<0.001
FEV_1_ (%)	60.18 ± 16.38	85.80 ± 7.42	<0.001

**Table 3:** Distribution of categorical variables in the total cohort and comparison between Group 1 and Group 2.

Variable	Category	Total Cohort	Group 1 (n = 257)	Group 2 (n = 133)	*p*-Value (χ^2^)
Sex	Male	207 (53%)	132	75	0.050
	Female	183 (47%)	125	58	
Living Area	Urban	216 (55%)	118	98	0.020
	Rural	174 (45%)	80	94	
Employment	Unemployed	164 (42%)	84	80	0.010
	Employed	207 (53%)	111	96	
	Retired	19 (5%)	18	1	
Education	Secondary School	212 (54%)	121	91	0.005
	High School	103 (26%)	68	35	
	University	74 (19%)	44	30	
	No Education	1 (0,3%)	1	0	
Presentation	Emergency	70 (18%)	64	6	0.030
	Outpatient	320 (82%)	193	127	
Smoking	Non-smoker	162 (42%)	117	45	0.010
	Ex-smoker	108 (28%)	91	17	
	Current smoker	120 (30%)	49	71	
Alcohol Use	Abstinent	202 (52%)	151	51	0.020
	Occasional	153 (39%)	60	93	
	Chronic	35 (9%)	10	25	

**Table 4:** Risk categories based on IPSS and clinical recommendations.

IPSS (Points)	Risk Category	Key Clinical Actions
<30	Low	Routine monitoring; conservative management
30–69	Moderate	Optimize inhaled therapy; offer psychological support; close follow-up
70–119	High	Multidisciplinary evaluation (pulmonology + psychiatry); escalate treatment
≥120	Very High	Aggressive management; consider hospitalization; rapid psychiatric review
Quartile-based stratification (for comparison in this cohort)		
≤17.79	Q1 (lowest quartile)	Routine monitoring; conservative management
17.79–35.91	Q2	Optimize inhaled therapy; reinforce self-management; consider psychological screening/support as needed
35.91–54.77	Q3	Closer follow-up; optimize therapy; consider multidisciplinary input if symptom burden or HADS is elevated
>54.77	Q4 (highest quartile)	Intensified management; prioritize multidisciplinary evaluation; consider early intervention pathways

Note: The pragmatic thresholds are intended for bedside usability and were not outcome-optimized. For comparison, we also report a distribution-based (quartile) stratification in this cohort (Q1 = 17.79; Q2/median = 35.91; Q3 = 54.77). Both pragmatic and quantile-based cut-points are cohort-dependent and warrant external recalibration and longitudinal validation.

## Data Availability

Data is contained within the article. The original contributions presented in the study are included in the article; further inquiries can be directed to the corresponding authors.
